# “They Love Their Patients”: Client Perceptions of Quality of Postabortion Care in North and South Kivu, the Democratic Republic of the Congo

**DOI:** 10.9745/GHSP-D-18-00368

**Published:** 2019-08-22

**Authors:** Julianne Deitch, Jean Pierre Amisi, Stephanie Martinez, Janet Meyers, Jean-Baptiste Muselemu, Jean Jose Nzau, Erin Wheeler, Sara E. Casey

**Affiliations:** a Reproductive Health Access, Information and Services in Emergencies (RAISE) Initiative, Heilbrunn Department of Population and Family Health, Mailman School of Public Health, Columbia University, New York, NY, USA.; b CARE Democratic Republic of the Congo, Goma, DRC.; c Save the Children USA, Washington, DC, USA.; d Save the Children Democratic Republic of the Congo, Goma, DRC.; e CARE USA, Atlanta, GA, USA.; f International Rescue Committee, New York, NY, USA.

## Abstract

**Background:**

Postabortion care (PAC) is a lifesaving intervention that, when accessible and of good quality, can prevent the majority of abortion-related deaths. However, these services are only sporadically available and often of poor quality in humanitarian settings. CARE International, the International Rescue Committee, and Save the Children strengthened the Congolese Ministry of Health to provide PAC, including voluntary contraceptive services, in North and South Kivu, DRC.

**Objective:**

We aimed to gain understanding of the demographic and clinical characteristics of PAC clients, the experiences of women who sought PAC at supported health facilities, and the women’s perceptions of the quality of care received. We also explored how client perspectives can inform future PAC programming.

**Methods:**

A PAC register review extracted sociodemographic and clinical data on all PAC clients during a 12-month period between 2015 and 2016 at 69 supported facilities in 6 health zones. In-depth interviews were conducted between September 2016 and April 2017 with 50 women who sought PAC in the preceding 3 months at supported health facilities. Interviews were recorded, transcribed, and translated into French for analysis. Thematic content analysis was subsequently used as the data analytic approach.

**Results:**

In 12 months, 1,769 clients sought PAC at supported facilities; 85.2% were at less than 13 weeks gestation. Over 80% of PAC clients were treated for incomplete abortion, and of these, 90% were treated with manual vacuum aspiration. The majority (75.2%) of PAC clients chose voluntary postabortion contraception. All but one interview participant reported seeking PAC for a spontaneous abortion, although most also reported their pregnancy was unintended. Clients were mostly made aware that PAC was available by community health workers or other community members. Experiences at the supported facilities were mostly positive, particularly in regards to client-provider interactions. Most women received contraceptive counseling during PAC and selected a modern method of contraception immediately after treatment. However, knowledge about different methods of contraception varied. Nearly all women said that they would advise another woman experiencing abortion complications to seek PAC at a supported health facility.

**Conclusions:**

The findings demonstrate the successful implementation of good-quality, respectful PAC in North and South Kivu. Overall, they suggest that the organizations’ support of health workers, including competency-based training and supportive supervision, was successful.

## Abstract (precis)

Women who sought postabortion care (PAC) at supported health facilities reported positive experiences, particularly regarding client-provider interactions, demonstrating the feasibility of implementing good-quality, respectful PAC in a humanitarian setting.

## INTRODUCTION

Among the 10 countries in the world with the highest maternal mortality ratios, 9 are affected by or emerging from war.^1^ Women and girls living in conflict settings have an increased need for sexual and reproductive health (SRH) services, while at the same time the availability of these services becomes limited.^2^^–^^4^ The collapse of health systems during conflict is often compounded by an emerging distrust in available services and health care providers.^3^^,^^5^ This barrier is particularly significant for services for which women and girls may face stigma from community members or health care providers, such as clinical management of rape, contraception, and care after unsafe abortion.^6^ Ultimately, women and girls living in conflict settings often face an increased risk of unintended pregnancy, unsafe abortion, and maternal mortality.^7^

Complications related to spontaneous or induced abortion account for an estimated 7.9% of maternal deaths globally and 9.6% in sub-Saharan Africa.^8^ Yet these figures are likely underestimations due to lack of reporting and misclassification of abortion-related deaths.^8^^–^^10^ The risk of dying as a result of spontaneous or induced abortion is significantly higher in settings with limited SRH services, and over 99% of abortion-related deaths occur in low- and middle-income countries.^8^ Further, where induced abortion is legally restricted or culturally stigmatized, women are more likely to resort to unsafe methods of induced abortion and may be less likely to access care for related complications.^11^

Postabortion care (PAC) is a lifesaving intervention that, when accessible and of good quality, can prevent the majority of abortion-related deaths.^12^ PAC includes 5 essential elements:

1. **Community and service provider partnerships** to prevent unintended pregnancies and unsafe abortions, to mobilize resources to ensure timely care for abortion complications, and to make sure health services meet community expectations and needs
2. **Counseling** to identify and respond to women’s emotional and physical health needs
3. **Treatment** of abortion complications
4. **Counseling and voluntary contraceptive services** to help women prevent future unintended pregnancies and abortions
5. **Reproductive and other health services** that are preferably provided on site or via referrals to other accessible facilities^12^^,^^13^

PAC is a lifesaving intervention that can prevent the majority of abortion-related deaths.

Despite the importance of PAC, such services are only sporadically available in humanitarian settings.^2^^,^^7^^,^^14^^,^^15^ Where services do exist, they are often of poor quality, and women face numerous barriers to receiving necessary care.^3^^,^^4^ Reviews of SRH services reveal that the quality of care is a critical factor in determining whether or not women seek care.^3^^,^^16^ Furthermore, evaluations of the quality of PAC services often rely on facility-level data, interviews with or observation of service providers, and client exit interviews.^17^^–^^21^ Rarely do studies seek a more in-depth understanding of client experiences with PAC services and their perceptions of quality of care, even though addressing client perspectives on quality of care has been shown to improve client satisfaction, leading to continued and sustained use of services and improved health outcomes.^22^ Further, evidence suggests that trust in the health system, via client-provider interactions and relations between a health facility and the community, for example, is an important element in restoring the social contract in a post-conflict setting.^23^ This article helps to address these gaps in the literature by demonstrating the feasibility of implementing good-quality, respectful PAC services in humanitarian settings, understood through the experience of PAC clients and their perceptions of quality of care.

## CONTEXT AND PROGRAM DESCRIPTION

The Democratic Republic of the Congo (DRC) has one of the highest maternal mortality ratios in the world, with 846 maternal deaths per 100,000 live births.^24^ Due in part to more than 20 years of conflict and instability, maternal mortality in eastern regions of the DRC is estimated to be higher than elsewhere in the country.^25^^,^^26^ Weakened health systems and limited health workforce capacity in North and South Kivu of the country have resulted in an inability to adequately respond to women’s health needs, including the provision of PAC services.^3^^,^^27^^,^^28^

Issues of availability and quality of PAC are compounded by a restrictive legal environment regarding induced abortion. The DRC has signed and ratified the *Protocol to the African Charter on Human and People’s Rights on the Rights of Women in Africa* (Maputo Protocol), a legally binding treaty that authorizes abortion in cases of “sexual assault, rape, incest and where the continued pregnancy endangers the mental and physical health of the mother or the life of the mother or the fetus.”^29^ As of March 2018, the DRC published the Maputo Protocol in the official journal, moving it closer to inclusion in national law. However, national legislation is generally interpreted to permit abortion only to save the life of a woman.^30^ Evidence suggests limited knowledge among both community members and health care providers on more detailed legal provisions.^31^ The DRC national health policy mandates the provision of PAC with manual vacuum aspiration (MVA) at health centers and hospitals, plus misoprostol at referral health centers and hospitals.^32^ However, misoprostol for PAC is not available at all mandated health facilities.

The Reproductive Health Access, Information and Services in Emergencies (RAISE) Initiative at Columbia University has collaborated with CARE International, the International Rescue Committee (IRC), and Save the Children to strengthen the capacity of the Congolese Ministry of Health (MOH) to provide PAC, including voluntary contraceptive services, in 4 health zones of North Kivu since 2011 and 2 health zones in South Kivu since 2008. This ongoing support to the MOH has included capacity building and supportive supervision of clinical providers, community mobilization activities, and provision of necessary equipment and supplies to facilities (Box).

BOX **Core Components of PAC Intervention**

- **Supplies and logistics:** Ensuring availability of MVA kits, misoprostol, medications (e.g. oxytocin, misoprostol), equipment, and contraceptive commodities
- **Strengthen skills of providers:** Competency-based training for appropriately skilled and supervised MOH health workers who respect privacy and confidentiality, provide full and accurate information, and ensure free and informed consent; values clarification and attitudes transformation
- **Supportive supervision:** Regular joint visits by the MOH and NGO staff to improve performance and ensure respectful care
- **Data collection:** Improving registers to collect appropriate information; training and empowering MOH providers to use data to improve their services
- **Community mobilization:** Outreach activities to conduct education in the community about danger signs during pregnancy and where to go for services and about contraception to respond to myths before they spread

Abbreviations: MOH, Ministry of Health; MVA, manual vacuum aspiration; PAC, postabortion care.

RAISE staff conducted baseline facility assessments (in 2007 in South Kivu and 2011 in North Kivu) that revealed weak PAC services across all 5 elements; MVA for PAC was not available at any facilities, and contraception, if available, was limited primarily to short-acting methods. In 2015, RAISE conducted program reviews that found that PAC was available and of good quality, but utilization of care was lower than expected. This raised questions about whether and to what degree services were underutilized and why. Accordingly, the partners conducted a mixed-methods program evaluation to better understand barriers and facilitators to accessing PAC services at health facilities, supported by the partner organizations in North and South Kivu.

This study aimed to understand the sociodemographic composition of PAC clients and, based on existing evidence of how quality of care influences utilization of PAC and other SRH services,^22^ to also understand the experiences of women who sought PAC at supported health facilities. More specifically, this analysis examines how client experiences with PAC can inform future programming. To our knowledge, this study is the first to look at client perceptions of PAC in a humanitarian setting.

## METHODS

### Study Design, Participants, and Data Collection

This article presents findings from 2 components of a mixed-methods study: a systematic PAC register review to understand the sociodemographic and clinical profiles of women who accessed PAC at supported health facilities and in-depth interviews (IDIs) with PAC clients to understand their perceptions of the quality of care provided at supported health facilities.

The PAC register review included data from all clients over a 12-month time period in 2015–2016 who received PAC at 45 health facilities in North Kivu, including 21 supported by CARE in Kayna and Lubero health zones, 24 supported by Save the Children in Masisi and Mweso, and 33 IRC-supported facilities in Kabare and Kalehe, South Kivu. Registers were developed in partnership with the provincial MOH, and each partner adapted the register slightly according to their project needs. Variables for each PAC client were extracted and included age, gestational age, parity, presenting complications, treatment received, and voluntary postabortion contraceptive uptake.

The IDIs were conducted between September 2016 and April 2017. Purposive selection of participants was conducted using PAC registers of 16 supported facilities, including 1 hospital, 5 referral health centers, and 10 health centers. To represent a range of ages, 8–9 clients per health zone (2–3 per facility) were selected. All participants had an MVA procedure for PAC at a supported facility within 3 months of the study date. Selected PAC clients were then contacted by a health worker and asked if they would speak to an interviewer about their experience at the facility. Health workers did not report refusals, and no women refused to participate once approached by the interviewer. A semistructured interview guide was developed by the research team.

Following the completion of a 5-day training on research ethics and methods, Congolese female facilitators conducted the IDIs in local languages (Swahili, Kinyarwanda, Kihavu, or Mashi). IDIs were held in a private room at the health facility to ensure the confidentiality of the discussion. Verbal informed consent was obtained from all participants. Participants were reimbursed for transportation between their home and the interview location. Interviews lasted 25–45 minutes. They were audio-recorded, transcribed, and translated into French for analysis. The researchers reviewed the French transcriptions to check the quality of translation and reverted back to the facilitators when clarification was needed.

### Data Analysis

The PAC register review data were entered into CSPro (version 6.3) and subsequently exported to SPSS (version 23) for cleaning and analysis. Using an inductive approach, the transcripts were first read by 2 researchers to identify overarching themes for the creation of a draft codebook, organized by general themes and subthemes. After discussing the draft codebook, electronic files containing the French transcripts were uploaded to NVivo (QSR International Pty Ltd) qualitative analysis software for coding. Several transcripts were coded separately by 2 researchers and the results were discussed to make revisions to the codebook, adding, deleting, or collapsing codes as necessary.

Once the codebook was finalized, thematic coding was independently performed in NVivo by 2 researchers. The consistency of coding was assessed by intercoder reliability, calculated as the number of agreements divided by the total number of agreements and disagreements. Disagreements were discussed and resolved until the interrater agreement was in the 90th percentile range. All transcripts were coded by 2 researchers, and selected transcripts were coded by a third researcher to ensure reliability and validity of the coding. Finally, the data were analyzed and presented using the respondents’ own words as illustrations.

### Ethical Considerations

Verbal informed consent was obtained from all participants. No participant names were recorded in the transcripts. Only study staff had access to the recordings. No personal identifying information was collected in the register review. Ethical approvals for the survey were obtained from the Columbia University Institutional Review Board and the Institutional Ethical Commission of the Catholic University of Bukavu (DRC).

## RESULTS

### Register Review

Over a 12-month period, 1,769 clients accessed PAC at 45 supported health facilities in 6 health zones. The average age of PAC clients ranged from 27.4 years old in Kayna/Lubero health zones to 29.3 years old in Kabare/Kalehe (Table 1). Only 10.3% of all PAC clients were under age 20 (data not shown). The mean parity of PAC clients differed significantly among health zones. In Kayna/Lubero, PAC clients had an average parity of 3.2, compared with 4.8 in Kabare/Kalehe and 5.0 in Masisi/Mweso. Similarly, in Kayna/Lubero, only 12.8% of PAC clients had 7 or more births, whereas in Kabare/Kalehe and Masisi/Mweso, 30.6% and 33.3% of PAC clients, respectively, had 7 or more.

**TABLE 1.** Postabortion Care Register Review Results

	**Total**	**Kayna/Lubero**	**Masisi/Mweso**	**Kabare/Kalehe**	***P* Value**
	**(N=1,769)**	**(n=477)**	**(n=644)**	**(n=648)**	
**Age, years, mean (SD)**	28.5 (7.3)	27.4 (7.0)	28.6 (7.2)	29.3 (7.5)	.001
**Age group, years, No. (%)**					
<25	570 (32.6)	186 (39.1)	192 (30.4)	192 (29.9)	
25–34	751 (42.9)	204 (42.9)	273 (43.3)	274 (42.6)	
≥35	429 (24.5)	86 (18.1)	166 (26.3)	177 (27.5)	
**Parity, mean (SD)**	4.4 (3.2)	3.2 (2.8)	5.0 (3.1)	4.8 (3.3)	.001
**Parity, No. (%)**					.001
0	169 (10.6)	80 (17.3)	31 (6.0)	58 (9.5)	
1–3	548 (34.4)	205 (44.4)	152 (29.3)	191 (31.3)	
4–6	456 (28.6)	118 (25.5)	163 (31.4)	175 (28.6)	
≥7	419 (26.3)	59 (12.8)	173 (33.3)	187 (30.6)	
**Gestational age, weeks, No. (%)**					.001
<13 weeks	1,331 (85.2)	416 (89.7)	414 (79.0)	501 (87.1)	
≥13 weeks	232 (14.8)	48 (10.3)	232 (21.0)	110 (12.9)	
**Method of treatment provided, No. (%)**	1,367 (81.3)	428 (93.9)	359 (61.3)	580 (90.6)	.001
MVA	N/A	408 (95.3)	340 (94.7)	No data	
Misoprostol	N/A	3 (0.7)	0 (0.0)	No data	
D&C	N/A	17 (4.0)	19 (5.3)	No data	
**Postabortion contraceptive use, No. (%)**					.001
No modern method	422 (24.8)	116 (24.8)	158 (24.7)	148 (24.8)	
IUD	513 (30.1)	145 (31.0)	104 (16.3)	264 (44.2)	
Implant	436 (25.6)	118 (25.3)	175 (27.4)	143 (24.0)	
Tubal ligation	2 (0.1)	1 (0.2)	0 (0.0)	1 (0.2)	
Injectable	228 (13.4)	42 (9.0)	151 (23.6)	35 (5.9)	
Pills	100 (5.9)	44 (9.4)	50 (7.8)	6 (1.0)	
Condoms	2 (0.1)	1 (0.2)	1 (0.2)	0 (0.0)	
**Facility type, No. (%)**					
Health center	1,055 (59.6)	96 (20.1)	395 (61.3)	564 (87.0)	
Referral health center	548 (31.0)	299 (62.7)	249 (38.7)	N/A	
Hospital	166 (9.4)	82 (17.2)	N/A	84 (13.0)	

Abbreviations: D&C, dilation and curettage; IUD, intrauterine device; MVA, manual vacuum aspiration.

Over a 12-month period, 1,769 clients utilized PAC services at 45 supported health facilities in 6 health zones.

The majority of PAC clients (85.2%) sought care prior to 13 weeks of pregnancy. Most PAC clients in all health zones had a presenting complication, with bleeding the most commonly reported. Yet in Kayna/Lubero, 74.7% had reported bleeding and 22.6% had no reported complications. In Masisi/Mweso, 51.2% had bleeding and 47.1% had no reported complications. In Kabare/Kalehe, complications were classified as severe (5.0%), mild (84.1%), or none (10.9%). (Specific definitions were not available for mild or severe complications. However, health workers described classifying an incomplete abortion without severe infection as a mild complication, and one with infection, severe bleeding, and other problems as severe.) In Kayna/Lubero and Kabare/Kalehe, over 90% of PAC clients received treatment for incomplete abortion, compared with only 61.3% of clients in Masisi and Mweso; most (nearly 95%) of these treatments were performed with MVA. Medications provided for PAC clients were noted only in 2 of the 3 program areas. Antibiotics were given to 62.6% (Kayna/Lubero) and 50.9% (Kabare/Kalehe) of PAC clients, and uterotonics to 46.3% (Kayna/Lubero) and 64.7% (Kabare/Kalehe). Pain medication was only recorded in Kayna/Lubero, where 23.4% of PAC clients received it.

Voluntary postabortion contraceptive use was reported for the majority of PAC clients (75.2%) in all health zones. Most PAC clients in Kayna/Lubero (56.5%) and Kabare/Kalehe (68.3%) chose a voluntary long-acting reversible contraceptive (LARC) method (intrauterine device or implant). In Masisi/Mweso, 43.7% of PAC clients chose a LARC, and 31.5% chose a short-acting method (pills or injectable). Across all health zones, young women under 25 years old were less likely to use modern contraception after abortion. Nearly 1 in 3 (29.7%) PAC clients under 25 did not choose a modern method, compared with 22.3% of women 25 and older. Less than half (48.5%) of women under 25 years chose a LARC, compared with 59.3% of women over 25 years.

75% of PAC clients in all health zones used voluntary postabortion contraception.

### In-Depth Interviews

#### Participant Demographics

A total of 50 IDIs were conducted with PAC clients who had an MVA at a supported facility: 34 in North Kivu and 16 in South Kivu. Participants were between 18 and 43 years old, and the majority were married (Table 2). Participants had varying levels of education. Most participants sought PAC during the first 12 weeks of pregnancy. Nearly half of participants had 7 or more pregnancies, and all had at least 1 prior pregnancy.

**TABLE 2.** Sociodemographic Characteristics and Reproductive History of Postabortion Care Clients Interviewed (N=50)

	**No.**
**Age group, years**	
18–24	8
25–34	22
35–49	20
**Marital status**	
Married	42
Unmarried	7
Unknown	1
**Level of education**	
None	16
Any primary	12
Any secondary or higher	21
Unknown	1
**Gestational age**	
First trimester	39
Second trimester	11
**No. of lifetime pregnancies**	
1–3	11
4–6	17
≥7	21
Unknown	1
**Postabortion contraceptive use**	
Long-acting method	20
Short-acting method	15
Modern method, unspecified	3
No modern method	12

#### Clients’ Perceptions of the Quality of Care Received

Clients’ perceptions of the care they received are presented below, by essential element of PAC.

**PAC Element 1: Community and Service Provider Partnerships to Prevent Unintended Pregnancies and Unsafe Abortions:** When asked about the pregnancy for which they sought postabortion care, most participants reported that it was unintended. For many, the age of their last child was cited as an indication of whether or not they were ready for another pregnancy.

I conceived when the baby was still very little, and I conceived without knowing, without wanting to. (26-year-old)

I had given birth to a new baby … a few months later, I noticed that I was pregnant again. After giving birth, the menstrual cycle normally stops … but me, I didn’t have that chance. (28-year-old)

Several women referred to the undesirability of having a baby due to displacement or poverty.

… among my 6 existing children, after so much displacement, at the time of conception I was feeling tired and asking myself how will I move and transport all those children, who were getting closer in age. (32-year-old)

No, I didn’t want any more [children] … I didn’t feel capable of handling another pregnancy, and I didn’t have the means to educate them or send them to school. (43-year-old)

All women except one reported that they sought PAC for a spontaneous abortion. The one participant who reported inducing abortion did so using quinine.

The interviews revealed widespread awareness of the availability of PAC but varying degrees of knowledge about specific services offered. Some women knew of services at supported facilities due to community mobilization activities, from community health workers and radio messages.

Interviews revealed widespread awareness of PAC but varying degrees of knowledge about specific services offered.

For this information, [community health workers] come to our house and ask us, each woman who is pregnant, to go quickly to the health center for care, because staying at home, many women die. (35-year-old)

… this message is even broadcast on the radio, they tell us that if a woman observes complications when she is pregnant, she must necessarily go to the health center. (43-year-old)

Many women knew of supported facilities since they went there for other health care for themselves or for their children. Others who were unaware of available services sought advice from family or friends, who informed them of the need to seek care and where to go.

When I told [my friend] about my problem, she said that she has no help to offer other than to tell me to go to the hospital. … That’s how I came here. (36-year-old)

**PAC Element 2: Counseling to Respond to Women’s Emotional and Physical Health Needs:** Participants reported overwhelmingly positive interactions with service providers at supported facilities, often citing their kindness and emotional support.

Participants reported overwhelmingly positive interactions with service providers at supported facilities.

They told me, Mama [Name], don’t be scared, you will be healed. It’s true, we know you feel pain, but it will pass. There is so much pain, but you will heal. (32-year-old)

Yes, [the provider] sat next to me, consoled me. You see, when you are ill, like for example, when I am sick … I can think that I will die. But since you are there, you take care of me, you sit next to me, you console me, telling me that I will not die, and me, I feel very at ease. (18-year-old)

Most women noted that they felt welcomed upon arrival at the facility and that providers neither discriminated against patients nor neglected them. Several women gave examples of how providers went above and beyond to care for them.

… she warmly welcomed me even though my condition was precarious. I had come with only one pagne [skirt], but she took courage to wash me and took her own pagne [skirt] to clothe me. (35 year old)

… a nurse bought me a Vital’o [soft drink] the day that I miscarried … I had some joy. And [she] bought me fruit. I was very happy. (22-year-old)

Not everyone had the same experience, however. The woman who reported an induced abortion indicated that providers were not entirely supportive.

… They asked me if I’m going to repeat this [act], and I said I will not. … they teased me, saying it’s not good. (18-year-old)

When asked if they believed that the information they shared with providers would be kept private, nearly all women expressed trust in the confidentiality of services. Many explained that maintaining privacy is the duty of health care providers. Further, several PAC clients commented on the lack of discrimination by the providers.

Because a nurse cannot reveal the secret, even if she saw a good or bad thing; she must keep it to herself. (40-year-old)

… the nurse should not disclose or reveal the secrets of the hospital. … according to medical ethics, no nurse or doctor has the right to disclose a patient’s information! (32-year-old)

We say that there is no discrimination here and that everyone is treated in the same way. The nurse is there to receive everyone. … Whether you’re Hutu, Hunde, or Tutsi, they should receive you. (36-year-old)

Participants were asked about the information they received during their visit and whether they felt that it was well explained. Several women described how providers framed ideas in a contextually accessible manner, passing along information with sensitivity.

[The provider] told me that I will be restored, I will be cured, and not to have bitterness about miscarrying, I will still be able to have another pregnancy. But he told me with how much I have suffered, I deserve care so that my health will recover so I can get pregnant again. (26-year-old)

Yes, we have discussed enough with him. He consoled me by helping me to understand what happened: it happened, better to hope for the next pregnancy and I should forget the past, and like this for 3 days, he comforted me until I was healed. (36-year-old)

Some, however, still had questions regarding their reproductive health. One explained how she remained uncertain as to the cause of her miscarriage.

The cause I cannot know, they often say it’s because the uterus is tired, but I also miscarried my first pregnancy; was the uterus already tired? Help me to understand if it gets tired, how? Because that’s what I’m often told. (32-year-old)

**PAC Element 3: Treatment of Abortion Complications:** The interviews with PAC clients suggested that treatments were effective and prevented any further complications. Women often spoke of feeling relief that they were cured, which was used as a qualifier of good quality of care.

Interviews with PAC clients suggested that treatments were effective and prevented further complications.

I felt free. After the miscarriage, they performed a good treatment, gave me a serum, and after having finished the infusion, I felt good. (36-year-old)

I thank God through the nurses for the medicines that I had. … before, when a woman aborted, she didn’t get better. But with these medicines, we have seen their effectiveness. This has helped us so much. (41-year-old)

Several women noted that, if not for the prompt services that they received, they would have died.

Without [the provider] I would have died, truly. … I would have died. When I arrived here, I was outside, then they went to look for him where he was treating other patients. When he realized that my problem was an abortion, he quickly left these patients to the other nurses to come take care of me, and immediately, he started treating me. (30-year-old)

They did something important for me, when I came, they welcomed me directly, they brought me to a place where they should receive me and treat me. … I appreciated that, [because] according to the situation in which I arrived here, if they hadn’t welcomed me well, I was going to die. (33-year-old)

Most women noted that the facilities were clean and well maintained, with adequate privacy. However, some negative experiences were mentioned in regards to overcrowding, including one woman who reported she was unhappy being placed in the postpartum ward after losing her pregnancy.

Some negative experiences were mentioned in regards to overcrowding and pain.

Why do they put the women who have aborted in the same room as the women who have babies? Normally they should separate them so that there is no confusion, also to not distress the women who have aborted. (32-year-old)

Other negative experiences described by participants were mostly related to pain from the procedure or lack of appropriate medications.

They cleaned me out. … my screams reached the road when I cried, they were doing the treatment; I felt a lot of pain. (22-year-old)

I felt a lot of pain during the treatment. I cried until people came to see, I was in a lot of pain, and the nurse told me to bear it. … I cried and asked him to give me anesthesia. He told me that they don’t provide anesthesia anymore, so I tolerated it. (36-year-old)

All participants were asked whether or not they paid anything when they received PAC. Most women in Masisi and Mweso stated that they had not paid for the care received, nor any component of contraceptive services. While some had sought assistance assuming they would have to pay, others had already been aware that services would be free due to outreach by community health workers and through word of mouth.

… and also they didn’t charge me money. I thought that we were going to pay even 5 or 10 dollars, but I left the hospital without paying; they told me the care is free. (22-year-old)

However, at facilities in Kabare, Kalehe, Kayna, and Lubero, nearly all women reported a cost associated with services, with most paying between US$8 and $15, and sometimes up to US$75. Some women believed they were paying directly for certain medications, while others were not sure what the cost was associated with.

This money, it’s not the nurse who keeps it, but it’s to buy medicines to help people. (41-year-old)

I think they took care of me and also bought the medicines, they paid the state taxes, that’s why I have to pay this money to the nurses. (32-year-old)

Several women highlighted how they were unable to pay the bill themselves and required assistance from friends, family, or community groups.

… the Church noticed that we weren’t able to pay the bill; my husband asked them to lend us the money. Since we were told to find half of the sum that they asked to release me and my child [who was ill at the same time]. The Church made a contribution of $30; we stayed with a debt of $20. … (35-year-old)

Despite having to pay for receiving care, many women believed that the cost was reasonable and were more than willing to pay when their health was at risk. A few women mentioned that they chose the supported facility because the cost was lower than elsewhere.

Despite having to pay for receiving care, many women believed that the cost was reasonable.

It [the cost] was not much compared to the medications I received. … And they did a great job cleaning my belly, giving me drugs. It [the cost] was really warranted. (32-year-old)

The price of the medication pushed me to come here because if I went to the general referral hospital, they would have to charge $50 or maybe $40, but here I don’t know if it’s their usual practice or not, I paid according to what women who abort pay. (30-year-old)

Moreover, women frequently explained that,even though they had to pay, they were first treated before being given a bill.

… it’s not just any nurse who can treat you without asking first for money. If he thinks of money, he can’t take good care of you. But also, I was in a critical state, if they neglected me, I was going to die. But despite my economic situation, they treated me. Yes, I would say that that they had the spirit of love. (35-year-old)

**PAC Element 4: Counseling and Voluntary Contraceptive Services:** Nearly all participants described receiving counseling on voluntary contraceptive services during their stay at the facility. For the most part, women understood that they could quickly become pregnant. The need to delay their next pregnancy was commonly described as giving their uterus a rest in order to improve their chances of having a future healthy pregnancy.

Afterwards they gave us instructions by showing that often when you abort, sometimes you get pregnant again directly after. This is why it’s better to protect myself by using family planning to recover. (33-year-old)

And she told me, Mama [Name], what do you think about it? Did you not risk dying? I told her that I had never used a contraceptive method like Depo or others, but this time, teach me if I can do something. (32-year-old)

Despite receiving contraceptive counseling, knowledge of the types of methods and their efficacy varied, and questions and worries regarding side effects often persisted.

Nearly all participants described receiving counseling on contraceptive services, but knowledge about methods varied.

If today at this very moment I decide to have this [method] removed, can they find it or not? And also, there are times that you want to get pregnant again, can they remove the [method] or can it disappear in the body and you become sterile—is this true or false? (22-year-old)

One often takes [the implant], it will disappear in the body, and then you find yourself in the hospital, the nurse looking for it unsuccessfully, you will be transferred to one of the big hospitals to undergo interventions. … Rather than use these things, it’s better to get pregnant and give birth. (20-year-old)

Several women’s comments suggested concerns about the quality of contraceptive services. For example, 3 women mentioned that the provider refused to administer a method without their husband present or requested their husband’s presence in order to receive a method.

[My husband] had categorically refused [contraception], so I began asking the doctor to give it to me in his absence. The doctor refused, saying that it could create problems for me at home. (22-year-old)

That’s when he told me about family planning. He told me about the methods and that I go tell my husband to write an authorization letter to allow them to give me the method. (40-year-old)

Ultimately, the majority of interview participants (38) reported that they chose a modern method of contraception at the time of receiving PAC. They expressed generally positive attitudes regarding their ability to delay or limit future childbearing.

Most interview participants reported accepting a modern method of contraception when receiving PAC.

In any case, this program that you’ve brought, it is a good program. Using family planning is very good because many carry pregnancies, especially the mothers you see that all have a pregnancy; a year passes; you have another pregnancy. … the other children are still very little, you ask yourself what to do since you lack [resources]. (28-year-old)

**PAC Element 5: Reproductive and Other Health Services:** Participants were not asked specifically about their need for reproductive and other health services, yet a few provided suggestions for how supported facilities may be improved. One woman mentioned the need for more specialized doctors to provide care for women.

I mean, if they can help us in settings like this, to find someone who can take charge, a medical specialist for women, who can help other general practitioners to treat women as necessary. (40-year-old)

Some participants were more specific in describing elements of care that were unavailable at the supported facility. For one woman, being transferred to another facility would have posed an unmanageable financial barrier.

Yes, even the bleeding continued and then the nurses … said that after trying and using all of the medicines, if it didn’t improve, they were going to transfer me to [Hospital]. … Unfortunately, I didn’t have money for treatment at the referral hospital or even for a transfer to another health center. (43-year-old)

Overall, women expressed that their health needs were met at the supported facilities. Often, this was relayed when they were asked whether or not they would recommend the facility to another woman who needed care. Overwhelmingly, participants were enthusiastic to explain how they would urge women to go to the supported facility, not only for PAC, but for other services as well.

Overall, women expressed that their health needs were met at the supported facilities.

I can inform her that [at this facility] they love their patients. They don’t neglect the patients. Sometimes there are hospitals that neglect their patients so that you don’t have the courage to send your friend or family. But if it’s a good hospital, you are encouraged to send your friends. (35-year-old)

## DISCUSSION

This study highlights women’s perceptions of the quality of PAC services in North and South Kivu, DRC, at health facilities supported by CARE, IRC, and Save the Children. The findings reveal that women who sought PAC services at supported facilities had extremely positive experiences, particularly in regard to client-provider interactions. The interviews also demonstrated an overall awareness of available PAC, suggesting that communication strategies are reaching communities as intended. The demographics of the interviewees were similar to the demographics of all PAC clients found in the register reviews, providing a means of triangulation of findings from both components of this study.

To our knowledge, this study is the first to explore how women perceive the quality of PAC in a humanitarian setting, and it fills a gap in the literature on the provision of PAC services in such settings.^4^^,^^14^ The findings from this study indicate that the program was successful in addressing the 5 components of PAC, particularly in regards to raising awareness of services and providing counseling, treatment, and postabortion contraceptive services. This demonstrates the successful implementation of good-quality PAC in a humanitarian setting. Similar to an evaluation of PAC services in Somalia, our findings suggest that a program approach of improved service delivery through capacity building of health providers and engagement of community members is successful and could be replicated in similar contexts.^14^ The interviews with PAC clients also suggest that perceptions of quality of care were not a significant barrier to seeking PAC.

The findings from this study indicate that the program was successful in addressing the 5 components of PAC.

The PAC register review findings are somewhat limited due to insufficient use of clear definitions for complications. Bleeding was recorded at high rates when providers recorded few serious complications, suggesting that any bleeding was marked as a complication. Additionally, antibiotics were often provided despite no record of infection. Both findings suggest a need to improve providers’ ability to accurately record complications and treatment. Program staff have noticed overadministration of antibiotics, and the problem is now discussed during supervision visits with providers. The register review also reveals that use of MVA for PAC, the standard of care recommended by the World Health Organization, and voluntary postabortion contraceptive uptake, including LARCs, were common. The lower percentage of PAC clients who did not receive treatment for incomplete abortion in Masisi/Mweso may be linked to the fact that 47% of women had no reported complications and interview participants from these health zones reported no cost for PAC services. Program staff believe women may be coming to supported facilities with miscarriages that do not require treatment.

The interviews highlighted the positive experiences women had with their care. Overwhelmingly, participants stated that they would recommend PAC to other women requiring care. Such statements were often a result of the woman herself being treated with kindness and dignity by the service provider. Further, supported facilities were often described as having a reputation of providing good-quality services, thus encouraging women to seek care for abortion complications. These findings are in line with existing literature on how quality of SRH services encourages care-seeking behavior.^22^^,^^33^^–^^36^ Research has also demonstrated that when providers are equipped with the tools necessary to deliver services and receive supportive supervision, they are more likely to treat clients with respect and take pride in their work.^37^

Participants stated that they would recommend PAC to other women requiring care.

While participants were satisfied with services, many reported pain during the procedure, suggesting the need to ensure that providers follow the pain management protocols on which they were trained. These include use of ibuprofen or paracetamol prior to the PAC treatment procedure and use of lidocaine and “verbacaine” or “vocal anesthetic” during the PAC treatment procedure. After the study, the MOH and NGO supervisors revisited pain management procedures with providers during supportive supervision visits.

Perceptions of quality of care as well as respectful care may be particularly relevant in humanitarian settings. Strengthening health systems that have been destroyed by conflict is essential not only to improve the health of the population but also to promote social cohesion and restore accountability.^23^ Trust in health care providers in North and South Kivu was apparent through women’s descriptions of their interactions with them, including with respect to confidentiality and nondiscrimination. The overall aim of these programs is to strengthen the capacity of the MOH to provide comprehensive SRH services. Client perceptions of PAC may very well translate into restored trust in the ability of the government to provide other health services.

Perceptions of quality of care as well as respectful care may be particularly relevant in humanitarian settings.

This study also provides insights into contraceptive knowledge, attitudes, and practices among women in the supported health zones. The majority of pregnancies ending in abortion were unintended, and the majority of women reported use of a modern contraceptive method following PAC, consistent with the register review findings that 75% chose a voluntary postabortion contraceptive. This finding indicates an acceptability of modern methods in the program areas, as well as a need for continued programming to increase awareness and access to appropriate contraceptive methods. Findings are in line with Demographic and Health Survey results that show high rates of unintended pregnancy and unmet need for modern contraceptive methods in North and South Kivu.^24^ The register review revealed that younger women are less likely to use a postabortion contraceptive, but it is unclear how much of this lower usage is due to client preference or provider bias. Contraceptive uptake among younger IDI participants was mixed, and thus their experiences did not help to answer this question.

Modern methods are acceptable in the program areas, but increased awareness of and correct information about appropriate contraceptive methods is needed.

Interviews with PAC clients revealed mostly positive attitudes toward contraception, yet attitudes were often undermined by confusion regarding method effectiveness and duration of use and by fears about side effects. While postabortion contraceptive counseling is one avenue of addressing these concerns, it is perhaps insufficient in providing a comprehensive knowledge of contraceptive methods and answering all questions regarding different methods. Several studies in similar contexts have found that postabortion contraceptive counseling leads to increased uptake of modern methods of contraception.^14^^,^^17^^,^^38^ Yet a systematic review of postabortion contraceptive counseling and services for women in low-income countries found insufficient evidence that postabortion contraceptive counseling leads to comprehensive knowledge of contraceptive methods or sustained contraceptive use.^39^

Notably, only one IDI participant reported seeking care after an induced abortion. It may be true that nearly all women sought care for a spontaneous abortion, but it is also possible that women decided not to disclose an induced abortion to the interviewers. Given the restrictive legal environment for abortion and strong social stigma, nondisclosure may be likely.^31^ Research in similar settings demonstrates that stigma is a significant barrier to care seeking for women who have induced abortion.^40^^–^^42^ The register review showed that, in all health zones, few nulliparous women sought treatment. This finding suggests that young unmarried women are less likely to seek PAC at supported facilities, perhaps due to fear of stigma. Common methods of inducing abortion in North and South Kivu are ineffective and often necessitate PAC services.^43^^,^^44^ Meeting the needs of women who have induced abortion is therefore essential in order to reduce abortion-related complications and mortality in North and South Kivu.

### Limitations

While the findings are mostly encouraging for the program, several key limitations may influence the reliability of the findings. The PAC register review data on complications and treatment were less consistent across partners and appeared to lack clear definitions, thus limiting their usefulness. Women who agreed to participate in interviews may have been those with a more positive experience at the facility, or women may have demonstrated a courtesy bias to please interviewers. Furthermore, only one woman reported seeking PAC after an induced abortion, which means we do not know about differences in quality of care for women who induce.

## CONCLUSIONS AND RECOMMENDATIONS

The findings from this study provide several key recommendations for future PAC programming, both in North and South Kivu and other humanitarian settings.

**Empower providers to provide good-quality care:** These programs have placed great emphasis on clinical training and ongoing supportive supervision, as well as working with providers to reinforce attitudes that promote good-quality care. These programming components empower providers to take pride in their services, and go above and beyond their duties to ensure that women are treated with care and dignity, as noted by all respondents.

**Provide ongoing support to ensure high-quality postabortion contraceptive counseling:** Providers require ongoing support to address the specific needs and challenges of PAC clients. During contraceptive counseling, providers should respond to any remaining concerns regarding the client’s method of choice. Developing materials for low-literacy populations that illustrate details on different contraceptive methods may help if women have follow-up concerns about their method of choice. Programs should also discuss provider attitudes that influence contraceptive care negatively, such as requiring a husband’s consent. More investigation is needed to assess whether provider attitudes are affecting lower uptake of postabortion contraception among younger women.

**Examine effective community mobilization strategies:** Community mobilization is a core component of the programs in North and South Kivu, and the findings reveal success in increasing community awareness of PAC. However, continued efforts are needed in raising awareness of the availability of contraception and dispelling myths surrounding various methods. Different strategies may be needed to serve young women, who were less likely to choose a postabortion contraceptive, and women who have induced their abortions, who may be less likely to seek PAC.

**Improve provider skills in pain management and accurate diagnosis and recording of complications:** The most commonly reported negative experience for PAC clients was related to pain during the procedure. Programs should reinforce provider skills in “verbal anesthetic” and use of recommended medications, such as nonsteroidal analgesics and ibuprofen, and should recommend that clinical supervisors observe whether these techniques are being used by providers. At the time of the study, few facilities offered misoprostol as an option for PAC treatment. As misoprostol becomes more available for PAC, women will have a choice for PAC treatment and should be informed of what pain to expect from each method in order to make their decision. Inconsistencies in the PAC register review suggest the need to also reinforce provider skills in diagnosing and recording abortion complications.

**Investigate costs paid by PAC clients:** PAC provided at the supported facilities is meant to be free of charge, yet women in some program areas consistently reported paying for some aspect of care. While cost did not appear to strongly influence client perceptions of quality of care, nor did providers delay treatment until payment was made, cost may serve as a barrier for women who wish to seek care but cannot afford to do so. Rates of treatment for incomplete abortion were higher in health zones where IDI participants frequently mentioned costs associated with PAC treatment, suggesting that women may be likely to only seek treatment when symptoms are severe. Partner organizations are currently investigating what costs are being paid for out of pocket by PAC clients at supported facilities.

**Further research to understand reasons for underutilization of PAC:** One underlying purpose of this study was to understand reasons for underutilization of PAC, and whether or not quality of care was a potential barrier. These interviews indicate that quality is not a barrier because the women reported overwhelmingly positive experiences at supported facilities. Further research is needed with women who do not come to supported facilities for PAC, and with women who may be more likely to experience poor quality of care, such as those who have induced abortion.

## Notes

**Peer Reviewed**

**Funding:** This study was funded by an anonymous private foundation.
